# Regulatory and Safety Requirements for Food Cultures

**DOI:** 10.3390/microorganisms5020028

**Published:** 2017-05-23

**Authors:** Svend Laulund, Anette Wind, Patrick M. F. Derkx, Véronique Zuliani

**Affiliations:** 1 Chr Hansen A/S, Bøge Alle 10, Hørsholm 2970, Danemark; dksl@chr-hansen.com (S.L.); dkawi@chr-hansen.com (A.W.); dkpde@chr-hansen.com (P.M.F.D.); 2 Chr Hansen SAS, Route d’Aulnay, Saint-Germain-lès-Arpajon 91180, France

**Keywords:** food cultures, regulation, safety

## Abstract

The increased use of food cultures to ferment perishable raw materials has potentiated the need for regulations to assess and assure the safety of food cultures and their uses. These regulations differ from country to country, all aimed at assuring the safe use of food cultures which has to be guaranteed by the food culture supplier. Here we highlight national differences in regulations and review a list of methods and methodologies to assess the safety of food cultures at strain level, at production, and in the final product.

## 1. Introduction

The fermentation of food of otherwise perishable raw materials has been used by man since the Neolithic period (around 10,000 years B.C.) [1]. Fast forward to today, where one-third of the human diet globally consists of fermented foods [2]. Thus the use of food cultures (FC) has increased significantly during the latter decades. Following this increase, guidelines dealing with the safety of FC have increased. In Europe, the General Food Law regulation [3] ensures a high level of protection of human life and consumers’ interests in relation to food. Other documents are also used as references regarding FC safety such as the Qualified Presumption of Safety (QPS) list from the European Food Safety Agency (EFSA) and the Inventory of Microorganisms with Technological Beneficial Use from the International Dairy Federation (IDF) [1]. FC suppliers have also developed strategies for testing FC on several criteria to assess the safety of the strains and their uses.

In the following, we will review the regulations developed in Europe and the tools available to specifically ensure the safety of FC. In that context, Table 1 presents the main entities and their respective roles in the FC safety evaluation process. We will also compare the European Union (EU) strategy with the regulatory framework in other parts of the world.

This publication does not cover the regulation of FC used as probiotics. The safety assessment is not different but the effects and claims are specifically regulated and listed for probiotics.

## 2. European Regulatory Framework

The use of microorganisms is one of the oldest food processing technologies resulting in the transformation and preservation of food. The formation of different metabolites contributes to the characteristic taste and texture. At the same time, it results in preserved and safe food with an extended shelf life. That is the reason why the category is regarded as a characteristic food ingredient forming part of fermented dairy, meat, bread, seafood, wine, cereals, vegetables, etc. products and thereby reducing the waste of many traditional foods. Despite FC being one of the world’s oldest categories of food ingredients, FC is not defined in EU legislation and this lack of a definition is the same for the majority of food legislation in the rest of the world. Within the EU, Denmark had national regulations requiring the approval of FC from 1973 until 2010. Voluntary notification and listing is still possible [4].

### 2.1. General Food Law: Obligation Concerns the Outcome and not the Mean. Food Including FC on the Market Must Be Safe

Food cultures (microorganisms) used directly in food production are regarded as food ingredients in the EU, a category of food ingredients with a very long history of use in a great variety of food products. FC, like other food ingredients, must fulfill the requirements set out in the General Food Law, EU Regulation No. 178/2002 Article 14 saying: Food shall not be placed on the market if it is unsafe and it is the food business operator’s responsibility for ensuring food safety [3]. FC used for the fermentation of food is not subject to EU premarketing regulation, unless it is regarded as being novel to the EU market and its consumers.

It is important to remember that foods are rich in microorganisms naturally present in and on the food. Spontaneous fermentation was the beginning of fermented food and still is a significant contribution to food. Even in ready-to-eat food products that are not regarded as fermented food, a hidden fermentation takes place by the indigenous flora during storage [5].

### 2.2. Specific Case: Novel Food

Novelty in this context means food not consumed to a significant degree within the Community before 14 May 1997 [6]. If that is the case, then the food has to comply with the updated Novel Food (NF) regulation EU 2015/2283 [7] repeating the aim from 1997 saying: “The novel foods and food ingredients concerned by this regulation are those which are not yet currently used for human consumption. This regulation applies to foods and food ingredients in the following categories: (…) foods and food ingredients which consist of microorganisms, fungi or algae (…)”. An NF has to undergo a pre-market evaluation and authorisation procedure including risk assessment by the EFSA and risk management by the EU Commission before it can be placed on the market.

Since 1997, no microorganism used and consumed as a live FC has been evaluated and authorised as NF in the EU.

A gene modification of a microorganism used as FC is also regarded as NF, but in that specific case the NF notification procedure is abandoned with respect to the genetically modified foods regulation and has to comply with EU directive No 2001/18 [8], EU regulation No. 1830/2003 [9], and Commission regulation No. 641/2004 [10]. No application has been made for the evaluation and authorization of any genetically modified FC in the EU.

## 3. EFSA and the QPS List

The Qualified Presumption of Safety list is the EFSA fast track risk assessment tool that is used by EFSA panels when evaluating products with microorganisms that require a premarket authorisation (e.g., feed additive cultures, cell factories producing enzymes/additives/vitamins, novel microorganisms, plant protection) [11]. It covers all risk assessment for microorganisms for human, animal, and environmental use—“from farm to fork”. It harmonises the work of the EFSA panels, makes the approach more transparent, improves the consistency of the assessments, and makes better use of resources by focusing on those organisms that present the greatest risks or uncertainties.

At EFSA, the BIOHAZ (Biological Hazards) Panel assesses the safety of biological agents taking into account:

- The definition of the taxonomic unit (establishing identity of the group)
- The body of knowledge
- The possible safety concerns (pathogenicity)
- For some species, the end use

**Figure 1:**
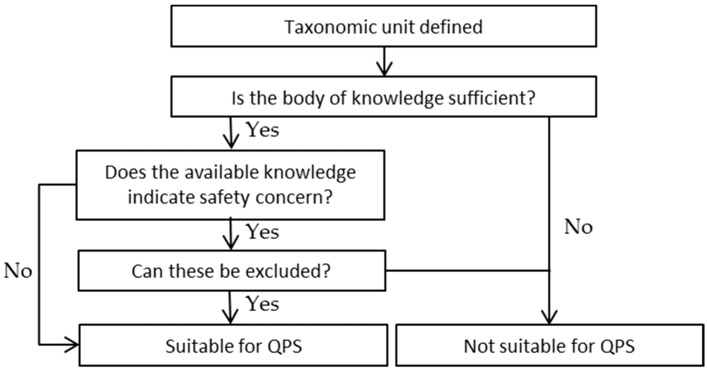
A generalised scheme for assessing the suitability for Qualified Presumption of Safety QPS status of a microorganism.

The scheme for assessing the suitability of microorganisms for QPS status is described in Figure 1 [12].

The introduction of a QPS list was made in 2007 as an opinion of the EFSA Scientific Committee after a public hearing [13]. It included species either presented to the EFSA or proposals made by stakeholders during a public consultation in 2005, even if they were not yet presented to the EFSA. The first QPS list consisted of 72 species (taxonomic units) with mutual or specific qualifications. The “Qualified” list included the examination of the lack of presence of virulence factors, toxic metabolites (*Bacillus*), resistance of some antifungals (some yeasts), and transmissible antibiotic resistance (bacteria) that have to be addressed on a strain-by-strain basis. In 2007, only one species had “intended use” as a qualification. In 2017, this had increased to eight species. For all the remaining species, safety applies to all uses of the species (taxonomic unit) and not to a product of or a product containing the microorganism. The last published update from March 2017 included 88 species [14]. Since 2014, filamentous fungi and enterococci have by default been excluded from QPS. At the same time, the requirement to EFSA was changed to updating of the list with new taxonomic units two times a year and then performing a total overall review of previously assessed microorganisms only every three years [11].

Microorganisms, which are not on the QPS list, are not necessarily considered to be unsafe. There are more reasons for not being on the QPS list, e.g., the species has not been evaluated by the EFSA, or it is not evaluated as safe independent of media, condition, and use, and would then remain subject to a full safety assessment for the particular use.

FC with a long history of safe use in food are considered as traditional food ingredients and are legally permitted for use in food in the EU without pre-market authorisation as described earlier. As a consequence, EFSA panels do not evaluate FC. Still, the QPS list can be consulted when safety evaluations of FC are made. After 10 years, the QPS list has created a certain reputation inside as well as outside the EU as “the positive list”, but this can create uncertainty regarding the use of FC which are not on the QPS list. EFSA’s application desk receives requests for the evaluation of FC species but EFSA has to reject these, as only the EU Commission can decide which microorganism applications require pre-market approval and safety evaluation.

## 4. EFFCA and the IDF Inventory

To fill the gap left by regulation, EFFCA (European Food and Feed Cultures Association) has proposed a definition of FC. The latest update from 2015 defines FC as “safe live bacteria, yeasts or moulds used in food production, and they are in themselves a characteristic food ingredient. FC preparations are formulations, consisting of concentrates (>10^8^ Colony Forming Units/g or mL) of one or more live and active microbial species and/or strains, including unavoidable media components carried over from the fermentation and components, which are necessary for their survival, storage and to facilitate their application in the food production process, and are in some cases standardised to a low count with carriers” [15].

With the EU Novel Food Regulation from 1997, microorganisms and fungi not yet currently used for human consumption are novel and must be regulated as such. With no list of microorganisms traditionally used in the EU before 1997, some doubt about the legality of FC produced and sold may emerge. To prevent the creation of uncertainty around the existing fermented dairy products, the FC, EFFCA, and IDF jointly produced “Inventory of Microorganisms with a documented history of use in food”, the first inventory of FC with a documented significant use in food production before 1997 [16]. The IDF/EFFCA inventory published in 2002 became a de facto reference for FC in practical use. This created the need for a list with a wider scope. With the 2012 inventory, an updated inventory of microorganisms used in food fermentations was published covering a wider range of food matrices (dairy, meat, fish, vegetables, cereals, beverages, and vinegar). Additionally, a review and update of the taxonomy of the microorganisms used in food fermentations were made in order to bring the taxonomy in agreement with the current standing in nomenclature. The inventory was expanded from 113 species in 2002 to 264 species in 2012 [2]. A more detailed description of the safety assessment, building of the list, and expected continued updates was published in The IDF Bulletin [17,18,19].

## 5. Other Available Tools to Assess the Safety of FC

Phenotypic methods to assess the safety of FC have been used for many years, and with the recent advances in molecular and nucleic acid-based methods and especially with the introduction of second-generation sequencing technologies, a multitude of new applications within the field of strain safety will improve the safety assessment of FC used in food.

### 5.1. Tools Used During the Screening

#### 5.1.1. Genome-Based Assessment of Food and Strain Safety

Whole Genome Sequencing (WGS) not only has the potential to replace conventional genetic fingerprinting techniques like Pulse Field Gel Electrophoresis (PFGE) [20], but it can also fill a caveat in strain safety assessment. Conventional strain safety assessments are based on culture-dependent methods like serotyping and phage typing or molecular techniques like PCR amplification and restriction digestion [21], and additional insights into the genetic basis of strain safety can be provided by WGS [22]. For this assessment, different tools are developed to screen for genomes with the presence of virulence factors and for antibiotic resistance genes. This includes the MvirDb database of microbial virulence factors [23] and the Virulence Factor Database [24], as well as the CARD database [25] and ResFinder for antibiotic resistance [26].

For the probiotic strain *Lactobacillus helveticus* MTCC5463, safety related genes were categorised into resistome, heavy metals, adverse metabolic genes, virulence related genes, and stress related genes [27], and the presence or absence of genes in these categories were used to aid the safety assessment of this strain. A similar approach was used for another gram-positive probiotic strain, *Bacillus coagulans* GBI-30, 6086 [28]. A more general approach, based on querying an assembled genome sequence with an in silico genome containing unwanted genetics, has the potential to be used as an in silico screen to select strains with a desired genetic signature [29].

A thorough taxonomical characterization is a prerequisite to use WGS as a tool to gain insight into strain safety, to assure a correct interpretation of the antibiotic resistance data, as intrinsic antibiotic resistance can be inherent in certain species of e.g., *Lactobacilli*, which are intrinsically resistant to vancomycin [30].

#### 5.1.2. Induction Test and Biogenic Amine Production

Healthy individuals rarely have adverse effects upon exposure to biogenic amines (BA) in concentrations normally found in fermented foods, since biogenic amines are effectively inactivated by the body. For example, histamine is effectively detoxified by the action of amine oxidases in healthy individuals. In individuals with certain genetic deficiencies or in treatment with drugs such as monoamino oxidase inhibitors (treatment of Alzheimer’s and Parkinson’s diseases) more or less severe toxic or allergy-like effects are seen when they are exposed to much lower concentrations of BA than the levels found in some foods.

As part of the screening for new strains for FC, a phenotypic assessment of the strains’ capability to produce BA needs to be performed in addition to the genetic screening for known genes involved in the formation of BA. The simplest way is to allow the strain to grow in an appropriate medium in the presence of the relevant amino acid precursors for the respective BA. The supernatants from such cultures are then assessed by HPLC for the presence of BA [31,32]. Only strains with no detectable BA-formation and with the absence of active genes known to mitigate BA formation should be selected for FC.

Formation of BA such as histamine, tyramine, putrescine, phenylethylamine, and cadaverine is well-known to occur in several fermented foods including fish, meat, wine, and cheese [33]. A comprehensive review of the scientific literature and risk of BA-formation in fermented foods was performed by the EFSA Panel on Biological Hazards (BIOHAZ) and was published in 2011 [34].

Food poisoning due to high concentrations of BA is usually caused by spoilage microorganisms that are unintentionally present in the food. In other cases it could stem from the FC if the strains in the culture have the ability to form BA. EFSA recommends good production hygiene combined with the use of FC confirmed as not producing BA and being able to outgrow any autochthonous microbiota under the conditions of production and storage [34].

#### 5.1.3. Test to Check Toxin Production (for Non-QPS Strains)

Many of the microorganisms used in food fermentation belong to species mentioned on the QPS list. Of the QPS listed species, a test for toxigenic potential is only required for *Bacillus*.

**Figure 2:**
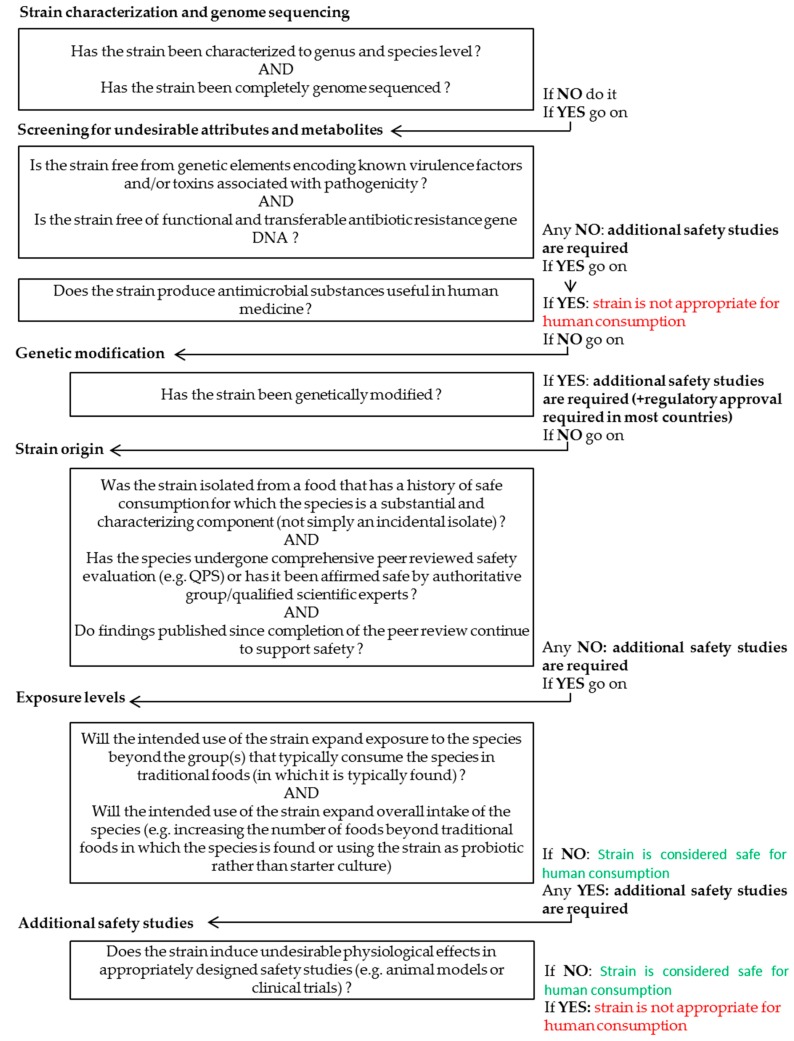
Decision tree for the safety assessment of microbial strains to be used in food applications [35].

For non-QPS species, a full safety assessment is needed before the microorganism is used as a FC. A general decision tree for safety assessment of both non-QPS and QPS strains to be used in food applications (Figure 2) has recently been proposed by Pariza et al. [35], and Bernardeau et al. [36] proposed a more specific decision tree for *Lactobacilli*. Proper species identification according to the current taxonomic opinion is crucial for determining whether the strain in question needs to be tested for virulence and toxigenic potential.

Among the non-QPS species commonly used in food fermentation are species belonging to genera such as *Staphylococcus* and *Enterococcus*. Some species in these genera are human pathogens such as *Staphylococcus aureus*. It is therefore natural to ask if strains in closely related species such as *S. carnosus* also possess a toxigenic potential. Apart from looking for potentially known toxin and virulence genes, phenotypic assessment is also needed because the genetic background for virulence and toxicity is far from being fully elucidated. Part of the phenotypic safety assessment of such microorganisms is to prove the absence of hemolytic and cytotoxic activity. The absence of hemolysis is most easily screened for by plating on blood agar.

A widely used method as a first screening for the cytotoxic potential of a microorganism is the application of sterile filtered culture supernatant to Vero cells in the Vero cell cytotoxicity test [37,38,39,40]. Microorganisms for FC should be non-hemolytic, negative in the Vero cell toxicity assay, and should not harbour any known active virulence or toxin genes.

#### 5.1.4. Antibiotic Susceptibility Tests

**Figure 3:**
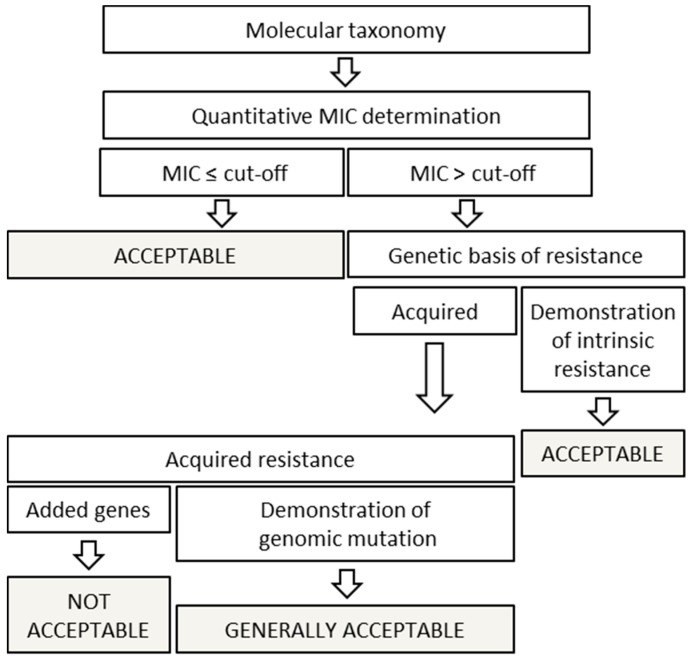
Proposed scheme for the antimicrobial resistance assessment of a bacterial strain (MIC: Minimal Inhibitory Concentration).

Individual strains of FC always need to be phenotypically assessed for susceptibility to clinically relevant antibiotics. Individual strains of FC as such are not pathogenic and thus do not need to be treated with antibiotics, but since they are alive when ingested in high numbers by the individual eating the food, they should not have antibiotic resistance genes that can be transferred to other (pathogenic) microorganisms in the gut and thereby making the curing of pathogens more difficult. Antibiotic resistance levels and the presence of transferable genes are well studied in many pathogenic species. The same is not true for the non-pathogenic FC species. The EU has published an antibiotic safety assessment tree that starts with phenotypic testing for susceptibility to a set of clinically relevant antibiotics together with guidelines for how to evaluate the test results according to so-called cut-off values [41], (see Figure 3). The cut-off values are set via data from available relevant published research papers, the European Committee on Antimicrobial Susceptibility Testing [42], and from national and European monitoring programs. For some microorganisms, they are given at the species level, for others at the genus level, and for yet others at the group level, e.g., obligate homofermentative *Lactobacillus*. This illustrates only that there is a lack of knowledge with regards to the normal antibiotic resistance levels within individual species which can to some degree make it difficult to get a proper, relevant antibiotic safety assessment of a given microorganism. In any case, the correct species identification according to current taxonomic opinion is a must for performing an appropriate evaluation of data for a given microorganism.

The assessment of phenotypic antibiotic susceptibility of microorganisms used in FC needs to be done by using the acknowledged methods that give minimal inhibitory concentrations (MICs) for the antibiotics for which the microorganism is tested [41].

Most individual strains used in FC belong to species that can be assessed by the method described in ISO 10932:2010 [43]—The method can be used for the determination of antibiotic susceptibility in *Bifidobacteria* and non-enterococcal lactic acid bacteria. For other species, the methods described by the Clinical and Laboratory Standard Institute [44] can be used.

The absence of phenotypic antibiotic resistance in strains used in FC is of course preferred, but should a resistance be observed, a proper analysis of the genome potentially combined with additional testing leads to the conclusion that the observed resistance is not transferable, and only then the strain(s) can be cleared to be safe for use in FC.

#### 5.1.5. Risk Assessment for the Safe Use of FC in a Food Application

Before the launch of a FC for a food application, it must first go through a simplified risk assessment procedure. It is a scientifically based process consisting of the following steps: (i) hazard identification, (ii) hazard characterisation, i.e., the evaluation of the nature of the adverse health effects associated with the hazard, (iii) exposure assessment, and (iv) risk characterisation. The risk characterisation represents the integration of the three previous steps to obtain a risk estimate, i.e., the likelihood and severity of the adverse effects which could occur [45].

In the context of FC used for a new application, the risk analysis focuses on the microbial hazard. The target is to evaluate whether the addition of a FC increases a bacterial hazard that has been identified in the manufacturing process of a given food to an unacceptable level or whether it brings a new hazard with an unacceptable exposure and/or unacceptable adverse health effect.

The intended use (FC dosage/mode of application/matrix in which it would be applied) and modification of the strain(s) exposure beyond the age group (consumer habits) are major parameters that have to be investigated and taken into account for the final safety evaluation of the new application. Thus in addition to the strain safety, the safe use of the strain is also checked.

### 5.2. Tools Used During Industrial Production

#### 5.2.1. Tools to Check Genetic Stability

When a strain has been selected through screening for the desired physiological properties, it has been species-identified according to the current taxonomic opinion and has been assessed to be safe for use. It is also important that the FC manufacturer can ensure and document that the strain remains the same each time the strain is produced. One way to ensure this is to have points 1–6 below in place to avoid genetic drift of the culture [46]:

1. Centralised in-house strain bank.
2. Reference stocks of the microorganisms should be stored below −80 °C to minimise changes to the strain that can occur during long-term storage. The temperature of the freezer should be under constant surveillance to make sure that the reference stock is kept at a constant temperature.
3. System for traceability.
4. All reference stocks should be recorded in a database where all retrievals or additions to the basic strain data record are also saved in log files. All inoculation material batches should be linked to this reference stock and all productions made with the inoculation materials should also be linked to the reference stock in order to give full traceability between the product and reference stocks.
5. Lowest number of generations from the reference stock to the final product. To minimise genetic changes happening from the reference stock to the final industrial scale batch of the strain, it is important to have a production process that secures the lowest number of generations possible between the reference stock and the final product batch. This can be done by making sure that each new production of inoculation material for the industrial scale production is started from the reference stock.
6. DNA fingerprint + basic phenotypic characteristic. Basic phenotypic analysis such as acidification profiles for strains used for yoghurt cultures or other phenotypic tests relevant for the specific microorganism should be part of the thorough characterisation done on each batch of inoculation material of the microorganism and compared to the phenotype of the reference material to ensure the stable performance of the microorganism.

Genotypic stability tests should also be part of a standard Quality Control (QC) analysis of new inoculation batches. Whole genome methods such as Pulsed Field Gel Electrophoresis fingerprinting or similar methods should be used to ensure that the inoculation material has exactly the same genomic profile as the strain’s reference stock material. Many FC strains have plasmids and important traits that might be encoded on the plasmids. It is therefore important to include tests for plasmid content as part of the QC on new inoculation batches to ensure that the plasmid content is the same as in the reference stock of the strain.

#### 5.2.2. Standard QC Analysis

Like other food manufacturers, FC suppliers have a strict focus on QC product safety at every stage in the value chain, from the raw material selection and surveillance of suppliers through production and packaging to distribution. That means that FC should rely on end product testing and on its process control. The ISO 27205:2010/IDF 149 on the standard of the identity of bacterial FC used in fermented milk products [47] give a description of the characteristics of bacterial FC regarding bacterial composition, cell concentration, contaminants, quality, and safety management. It also provides a list of methods of analysis to assess compliance. EFFCA has also developed guidelines for its members to harmonise QC of meat cultures [48]. Lastly, although not standardised internationally, equivalent QC procedures are also used for FC produced for other food applications.

As FC are applied in food and beverages, FC production has to comply with food safety standards. The implemented quality system combines Good Manufacturing Practices (GMP) for food and Hazard Analysis of Critical Control Points (HACCP). The HACCP principles are based upon:

- Identification of potential contaminants
- Definition and mapping of critical control points (CCPs) and operational Pre-Requisite programs (OPRPs)
- Definition of critical limits for CCPs
- Scheduling and reporting of measurements and observations
- Procedures for corrective actions when monitoring deviations and critical limits
- Procedures to verify the effectiveness of the HACCP plan

Microbial criteria and specifications for process hygiene and food safety criteria have been set up in order to define the acceptability of the processes and the final products. Every single batch of FC is microbiologically controlled. According to EC N° 2073/2005 [49] on the microbiological criteria for foodstuff, FC have to be free from *Salmonella* and *Listeria monocytogenes* in 25 g. Contamination with other microorganisms is also checked, and targeted microorganisms and methods vary from end product to end product, e.g., taking into account food applications and nature of the species included in the FC, among others. In addition to analysing the finished goods, bulk and environmental samples are also analysed on a regular basis. When available, international standard methods should be used.

## 6. Regulatory Framework outside the EU

Many countries have no product specific legislation while others do have specific legislation and/or standards for fermented food products that may regulate the use of FC.

Like the EU, the USA has no specific regulation for FC. Some species are regarded as “safe and suitable” for human consumption while others have a status as Generally Recognized As Safe (GRAS) and are notified to the Food and Drug Administration (FDA) and published [50]. “GRAS self-determination” is a third status conducted by the individual supplier. The “GRAS self-determinations” are not public, and consequently, no overview is possible [51]. Safe or safety means that there is a reasonable certainty in the minds of competent scientists that the substance is not harmful under the intended conditions of use. However, it is impossible in the present state of scientific knowledge to establish with complete certainty the absolute harmlessness of the use of any substance. Safety must be determined by the published general recognition of safety. There are several differences between GRAS and QPS:

- GRAS is for all ingredients
- QPS is for microorganisms only
- A GRAS FC is at the strain level and for a particular food product
- The QPS status applies to the taxonomic unit of a species of microorganisms and not to the product containing the microorganisms

The safety issues for GRAS and QPS will therefore be different. A more detailed overview of the differences is described in a scientific opinion by EFSA [11]. Feed cultures are regulated and approved separately from FC by the FDA.

In Japan in general, all lactic acid bacteria are allowed as FC. They are regarded as food but in terms of regulations they belong to the “List of substances which are generally provided for eating or drinking as foods and which are used as a food additive” [52] as “Lactic acid bacteria concentrates”. Some countries have established positive lists for FC. Examples are Thailand, China, and Malaysia. In Thailand, industrial FC are regarded as food additives. In order to be listed as permitted lactic acid bacteria for the fermentation of dairy or beverage products, the legal framework requires the application of documents on the strain level for registration to the Food and Drug Administration [53]. Traditional fermented foods (spontaneous fermentation) without the use of FC are outside the registration requirements.

Since 2010, China has had a specific regulation for FC. FC that had a history of traditional use could be continually used after 2010. For the time being, no list of traditional FC species used in China is available. Works have been ongoing for the last few years to build it, and the final list of species is expected to be published in the near future. New FC shall go through a Novel Food Material Application on the species level. From 2010 and as of May 2017, 35 species are approved for use in fermented food products. The official list [54] has not been updated since August 2014 and includes only 29 species.

Malaysia has in its Malaysian Food Act from 2014 a “New Regulations 26B” that has the legal framework for “Microbial cultures for food fermentation” [55]. Other countries have import/product registrations (Argentina, Colombia, Mexico, Vietnam, Indonesia, Egypt, Iran, Jordan, Lebanon, Libya, Saudi Arabia, United Arabia Emirates, and Russia-Kazakhstan-Belarus (RU Customs Union).

In the Russia-Kazakhstan-Belarus Customs Union, FC are regarded as food and the Technical Regulation “On Safety of Milk and Dairy Products” (TR TS 033/2013) [56] is the key regulation covering the standards and requirements for milk and dairy products including the use of FC. Manufacturers of dairy products shall ensure safety of an industrial FC and processes of its manufacture, as well as its compliance with the requirements of the document.

The Euro-Asian Council for Standardization, Metrology, and Certification is for the time being drafting general specifications for “Bacterial Starters for Dairy Products” expected to be official starting from 2017. The Euro-Asian Council covers the following countries: Azerbaijan, Armenia, Belarus, Georgia, Kazakhstan, Kyrgyzstan, Moldova, Russia, Tajikistan, Turkmenistan, and Uzbekistan. The draft is expected to be close to the present Russian Custom Union rules.

## 7. Conclusions

FC, similar to all other ingredients used in foods, need to be safe. In the EU this is set out in the General Food Law. In this context, FC suppliers have an obligation with respect to the results. The means to reach this safety target are mainly the tools used by other food ingredient manufacturers: HACCP and good manufacturing practices such as sampling of the environment and the ingredients on a regular basis. On top of these basic control and monitoring systems, FC suppliers also have to consider the specificity of this ingredient. FC are live bacteria. EFSA, IDF, and EFFCA have proposed additional tools and methods to evaluate the safety of FC with the unique target of keeping a high level of food safety and to protect human life and health. FC suppliers have also implemented control points to evaluate if the FC produced are safe and used in a safe way.

In the rest of the world, the method of handling FC safety is sometimes significantly different. As an example, the GRAS status from the US is often not properly understood in the EU and brings confusion, particularly when considering the purpose of the QPS list or the IDF inventory. Another example is that in several countries in Asia, deliberately added industrial FC are not regarded as food ingredients but as food additives and therefore have to undergo registration, including safety evaluation, before being marketed. In contrast to this, in the same countries there is no regulation of FC used in food, which in the individual countries is seen as traditional food production.

The safety of FC is guaranteed thanks to approaches that may differ from country to country. Nevertheless, whatever the strategy applied, it is imperative to have an evaluation of the FC safety at three levels:

- At the strain level.
- During production.
- In the process it is applied to and throughout the shelf life of the food.

## Figures and Tables

**Table 1:** Role of the main entities involved in the food cultures (FC) safety assessment in the European Union (EU).

**EU Commission**	The European Commission is responsible for creating the General Food Law and for risk management (policy) of safety systems together with the Member States.
**EFSA**	The European Food Safety Authority is an agency funded by the European Union that operates independently of the European legislative and executive institutions and EU Member States. EFSA is responsible for the risk assessment.
**FEEDAP**	The EFSA Panel on Additives and Products or Substances used in animal feed provides scientific advice on the safety and/or efficacy of microorganism-based additives and products or substances used in animal feed.
**BIOHAZ**	The EFSA panel on Biological Hazards provides scientific advice on biological hazards in relation to food safety and microbiological criteria.
**IDF**	The International Dairy Federation represents the global dairy sector and ensures that the best scientific expertise is used to support high quality milk and nutritious, safe, and sustainable dairy products.
**EFFCA**	The European Food and Feed Cultures Association cooperates, both within the EU and globally, with a wide range of stakeholders in the area of microbial FC.
